# The Adult Ts65Dn Mouse Model of Down Syndrome Shows Altered Swallow Function

**DOI:** 10.3389/fnins.2019.00906

**Published:** 2019-08-29

**Authors:** Tiffany J. Glass, Luke Carmichael V. Valmadrid, Nadine P. Connor

**Affiliations:** ^1^Department of Surgery, University of Wisconsin–Madison, Madison, WI, United States; ^2^Department of Communication Sciences and Disorders, University of Wisconsin–Madison, Madison, WI, United States

**Keywords:** deglutition, deglutition disorders, swallow, Down syndrome, mouse, VFSS, Ts65Dn, Dp(16)1Yey

## Abstract

There are increased risks for deglutition disorders in people with Down syndrome (DS). Although mouse models have been used to study the biological underpinnings of DS in other areas, relatively little is known about swallowing phenotypes in these models. We hypothesized that swallowing performance would be affected in adult mouse models of DS, relative to typical control mice. Videofluoroscopic swallow studies (VFSS) were conducted on adults of two mouse models of DS: Ts65Dn and Dp(16)1Yey, and evaluated in comparison with age-matched controls. Relative to other groups, adult Ts65Dn showed significantly slower swallow rates, longer inter-swallow intervals (ISI), and greater numbers of jaw excursion cycles preceding each swallow. In contrast, adult Dp(16)1Yey mice showed swallowing performance similar to control mice. Exploratory quantitative analyses of the intrinsic tongue (transverse muscle), and extrinsic tongue muscles [genioglossus (GG), styloglossus (SG), and hyoglossus (HG)] showed no significant differences between genotype groups in myosin heavy chain isoform profiles. Collectively, these findings suggest that while swallowing is typical in adult Dp(16)1Yey, swallowing in adult Ts65Dn is atypical due to unknown causes. The finding that adult Ts65Dn may have utility as a model of dysphagia provides new opportunities to elucidate biological underpinnings of dysphagia associated with DS.

## Introduction

Down syndrome (DS), typically caused by a trisomy of the 21st chromosome, is associated with increased risks for feeding challenges and deglutition disorders across the lifespan (Lazenby, 2008). These can coincide with medical comorbidities (Dinan and Golden, 1990; Zárate et al., 2001; Prasher et al., 2004; Abanto et al., 2011), craniofacial differences (Hashimoto et al., 2014), sensory differences (Frazier and Friedman, 1996), and behavioral differences (Homer and Carbajal, 2015). In patients with DS in which impairment of deglutition is suspected, videofluoroscopic swallow studies (VFSS) may detect swallowing impairments (O’Neill and Richter, 2013; Jackson et al., 2016). While clinical studies have reported both oral motor dysfunction and pharyngeal phase dysphagia in children with DS (Jackson et al., 2016), it is also known that adults with DS are likely to experience several additional risk factors for swallowing disorders (Lazenby, 2008). Dysphagia associated with DS in later life stages can coincide with multiple challenges including an increased prevalence of age-related comorbidities (Yang et al., 2002; Cipriani et al., 2018), unique health care and support needs (Carling-Jenkins et al., 2012; Capone et al., 2018), and communication impairments associated with the syndrome that may obstruct self-reporting of symptoms (Lazenby, 2008; Capone et al., 2018). One avenue for addressing this research challenge may be the use of animal models, which offer opportunities to explore aspects of pathophysiology of feeding and swallowing that cannot be studied in patient populations (German et al., 2017).

Recent work has refined the application of experimental VFSS for the quantitative study of swallowing performance in animal models of swallowing disorders. For example, VFSS has been used to detect and characterize swallowing differences in rat models of aging and Parkinson disease (Russell et al., 2013; Cullen et al., 2018), mouse models of presbyphagia (Lever et al., 2015b), and ALS (Lever et al., 2010). This validated experimental paradigm offers new opportunities for similar evaluation of swallowing differences in mouse models of DS. The Ts65Dn and Dp(16)1Yey mouse models of DS are both used for translational research (Liu et al., 2011). While Ts65Dn models DS through a partial trisomy comprised of a distal portion of mouse chromosome 16 and a centromeric portion of mouse chromosome 17 (Davisson et al., 1993; Duchon et al., 2011), Dp(16)1Yey features a typical karyotype with a duplication of the entire mouse chromosome 16 region that is syntenic to the human 21st chromosome (Li et al., 2007). While these models are generally regarded as having utility for the study of biological differences associated with DS, relatively little is known about their feeding and swallowing phenotypes.

Our prior work suggested normal swallowing performance in juvenile (5–6-week-old) Ts65Dn mice, as assessed by swallow rates (Glass and Connor, 2016). However, because impairments associated with DS are known to change with age (Bayen et al., 2018), we hypothesized that swallowing performance would be affected in adult mouse models of DS. We report the outcomes of VFSS and initial tongue muscle characterization of two adult mouse models of DS: Ts65Dn and Dp(16)1Yey.

## Methods

### Mice

All husbandry and experimental procedures involving live mice were carried out with approval from the University of Wisconsin School of Medicine and Public Health Animal Care and Use Committee (IACUC) and were conducted in accordance with the Guide for the Care and Use of Laboratory Animals (8th ed.) (The National Academies Press, 2011). Mice were weaned at 3 weeks of age and raised on a hard pellet diet. Ts65Dn were maintained from The Jackson Laboratory mouse stock number 005252, by pairing Ts65Dn females with B6EiC3Sn.BLiAF1/J males (stock number 003647). Dp(16)1Yey mice were assessed in the same genetic background, as achieved through multiple backcrosses to B6EiC3Dn.BLiAF1. Dp(16)1Yey used in the present study were generated by mating Dp(16)1Yey females to B6EiC3Sn.BLiAF1/J males. Genotypes were determined by separated PCR or by Transnetyx as previously reported (Glass and Connor, 2016).

Mice were analyzed in videofluoroscopic swallowing assays in comparison to age-matched euploid sibling controls (Ts65Dn) and WT sibling controls [Dp(16)] at 5 months (19–22 weeks) of age (9–13 mice per group). This is an age immediately preceding the onset of age-related neurodegeneration and associated cognitive decline reported to occur in Ts65Dn at 6 months of age (Holtzman et al., 1996; Hunter et al., 2003). These groups subsisted on Harlan Teklad diet 7913, which is a hard food pellet. Separately, male and female Ts65Dn mice were evaluated in videofluoroscopic swallowing assays in comparison to euploid controls, in groups comprised of mice spanning an adult age range (9–36 weeks of age, 7 mice per group). This age range encompasses early adulthood through late maturity, and was anticipated to provide a preliminary indication of whether swallowing phenotypes differed with sex and age. Video-recording assays of mastication of hard food pellets were also performed on male mice across an adult age range (8–44 weeks of age, 13–14 mice per group). All age range groups subsisted on Harlan Teklad diet 8604, which is a hard food pellet. Following behavioral experiments, mice were weighed, euthanized with CO_2_, and muscles were isolated and stored at −80°C for subsequent analysis.

### Videofluoroscopic Swallow Studies

Videofluoroscopic swallow studies assays were performed as previously described (Glass and Connor, 2016). Mice were identified by alphanumeric codes to mask genotype identities to workers during VFSS assays. The food used for imaging was a 2:1 mixture of Fritos Mild Cheddar Cheese^TM^ dip and 40% weight/volume barium suspension. Mice were acclimated to cheese mixtures daily for a few days immediately prior to VFSS, and all food was withheld overnight prior to the VFSS study. On the day of data acquisition, each mouse was placed alone in a cage and was imaged during continuous volitional eating at 30 fps with an Artis Zee system (Siemens Healthcare, Forchheim, Germany). The duration of VFSS acquisition for each mouse was limited to the amount of time required to record several visible swallows, typically at least 10 s. For all experiments, each mouse generated VFSS data for only one time point. Following VFSS, mice were weighed.

### Videofluoroscopic Analysis

Videofluoroscopic swallow studies footage was manually analyzed in ImageJ using measures as previously described, to quantify measures of oral and pharyngeal function (Lever et al., 2015a, b). These measures included swallow rate (the number of swallows per 2 s of eating), inter-swallow interval (ISI, the number of seconds elapsed between two successive swallows), jaw excursion rate (JER, the number of cycles of jaw opening and closing per second), and jaw cycle:swallow ratio (JSR, the number of jaw excursion cycles per ISI). In these studies of a puree consistency, the JSR measure is analogous to the lick-swallow ratio (LSR) previously described in analysis of drinking (Lever et al., 2015a, b), however, in the present study the JSR acronym was used to accommodate the possibility that with a puree consistency jaw cycles may indicate other oral processing movements and/or licking. These measures were obtained using a minimum of three and a maximum of five separate measurements for each mouse, which were then averaged to generate each final data point. Intrarater and interrater reliability for this study were evaluated through interclass correlation coefficients (ICC). Values of 0.90 or greater were obtained for all measures, which indicated robust agreement for evaluation of VFSS (Nordin et al., 2017).

### Mastication Rate Analysis

To supplement VFSS analysis which evaluated swallowing performance during consumption of puree, high-speed videorecording was used in separate sessions to evaluate mastication of a hard food pellet. Food was removed from the cage the evening prior to the assay and restored the following morning at the start of the assay. Mice were videorecorded at 60 fps in lateral profile while eating. Video footage was manually analyzed in Adobe Photoshop CC to quantify mastication rate as previously described (Lever et al., 2009). The analysis began on a frame with the jaw maximally closed, and the rater counted the number of subsequent mastication cycles (jaw fully open and then closed) that occurred during the next 59 consecutive frames. This provided the number of chewing cycles per second. The mastication rate for each mouse was determined from the average of four to five different 1-second periods of continuous chewing.

### Immunofluorescence and Microscopy of Intrinsic Tongue Muscles

Intrinsic tongues were examined through tissue sections due to the unique anatomical specialization and complex interdigitation of these muscles (Cullins and Connor, 2017). Tongues of adult mice were embedded in OCT (Optimum Cutting Temperature) in plastic cassettes, and frozen through immersion in isopentane cooled in liquid nitrogen. Tissue was sectioned at 10 μM onto slides and unfixed tissue sections were incubated overnight at 4°C with primary antibodies mouse IgG1 SC-71 (applied at 1:1), mouse IgM BF-F3 (applied at 1:1; Developmental Studies Hybridoma Bank), or rabbit anti-Laminin (applied at 1:200; Sigma, L9393), to detect myosin heavy chain isoform (MyHC) 2a, MyHC 2b, and myofiber borders, respectively. In each technical staining iteration, mouse soleus and extensor digitorum longus (EDL) muscle sections were processed concurrently as biological positive and negative controls for MyHC isoform staining. After PBS washes, slides were incubated for 1 h at room temperature with fluorescent secondary antibodies from Thermo Fisher Scientific (goat anti-mouse IgM AF350, A31552 (1:50), donkey anti-rabbit IgG AF488 A21206 (1:1000), goat anti-mouse IgG1 AF568, A21124, 1:300), rinsed, mounted, and imaged. Imaging was performed on an epifluorescence Olympus Bx53 microscope with a motorized stage and CellSens software. Optimal image exposure settings for all isoforms were experimentally determined for each image acquisition session through evaluation of biological positive control staining for MyHC 2b (EDL muscle), biological positive control staining for MyHC 2a (soleus muscle), biological negative control staining for MyHC 2b (soleus muscle), and technical negative control slides with all primary antibodies omitted to control for autofluorescence. Tissue sections were imaged through semi-automated acquisition to generate composites of multiple fields of view acquired with a 20× objective. One large composite image was obtained from one section from each intrinsic tongue.

### Quantitative Image Analysis

Quantitative analyses of myofibers of the transverse intrinsic tongue muscle were performed on images cropped to isolate one large region of interest within the blade of the tongue. Analyzed regions were located within an area bordered anteriorly by the tongue tip, posteriorly by the frenulum (posterior limit of ventral tongue mucosa), superiorly by the superior longitudinal (SL) muscle, and inferiorly by the inferior longitudinal (IL) muscle (Figure 3A). Regions of interest were analyzed through the MATLAB Application SMASH as previously described (Smith and Barton, 2014). Laminin staining was used for the semi-automated detection of myofibers, allowing myofiber size to be quantified. MyHC signal was used to calculate the percentage of fibers positive for each isoform. At least several hundred myofibers located in the blade of the tongue were analyzed to produce one average data point for each mouse.

### MyHC Protein Analysis of Extrinsic Tongue Muscles

Total protein was isolated from homogenized extrinsic tongue muscles of adult mice using methods previously described (Adreani et al., 2006). Following sample protein quantification through a Bradford protein concentration assay (Thermo Fisher Scientific), MyHC profiles of extrinsic tongue muscles were analyzed through separation by sodium dodecyl sulfate polyacrylamide gel electrophoresis (SDS-PAGE) (Adreani et al., 2006), and silver staining to visualize protein isoform bands. Stained gels were digitally scanned using a flatbed scanner and band signal intensity was quantified using UN-SCAN-IT gel analysis software (Silk Scientific). Each mouse muscle sample was analyzed in duplicate.

### Statistics

GraphPad Prism 7.04 was used to perform one- or two-way ANOVAs as applicable, with Tukey’s *post hoc* tests. One-way analysis of variance was used to evaluate the impact of genotype (euploid, Ts65Dn, WT, and Dp(16)1Yey) on swallowing measures for male 5-month-old mice, and two-way analysis of variance was used to evaluate the impact of genotype (euploid, Ts65Dn) and sex (male, female) on swallowing measures for groups comprised of Ts65Dn mice and controls spanning the adult age range. An unpaired *t*-test was used to evaluate mastication rates in male Ts65Dn and male euploid controls. IBM SPSS Statistics software was used to calculate ICC values to evaluate VFSS interrater and intrarater reliability. An α-level of 0.05 defined statistical significance.

## Results

### VFSS

#### 5-Month-Old Ts65Dn and Dp(16)1Yey

**FIGURE 1:**
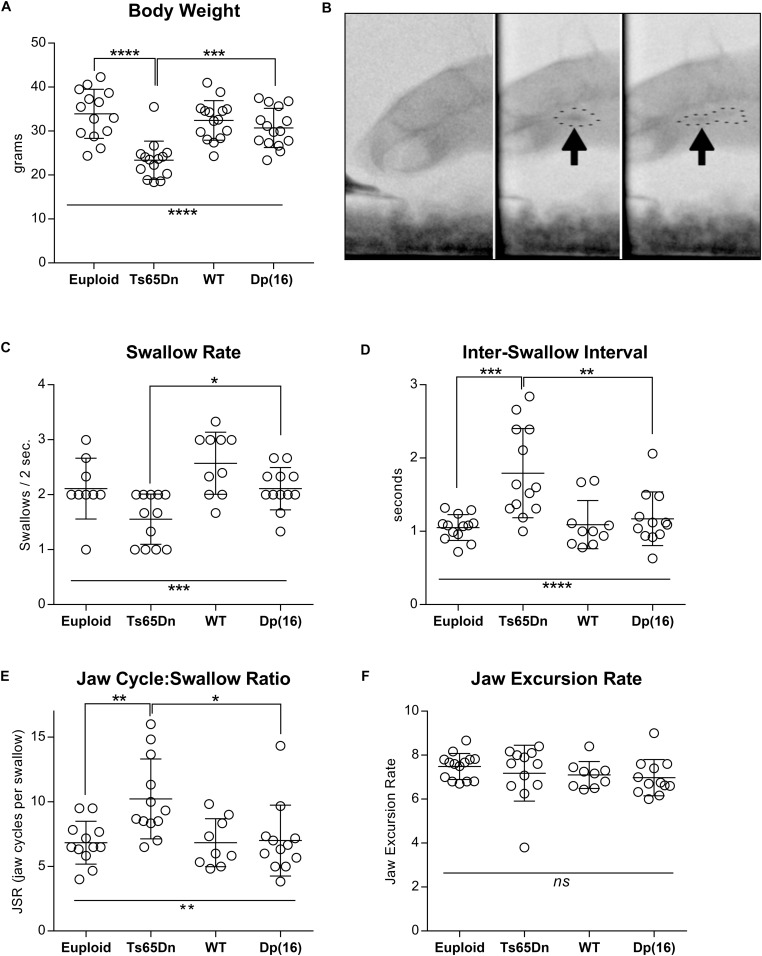
Videofluoroscopic swallow studies analysis in 5-month-old male Ts65Dn and Dp(16) mice. **(A)** Total body weight of mice analyzed through VFSS. **(B)** Stills from VFSS footage of adult mice. Arrows and dotted outlines show a food bolus immediately prior to initiation of a swallow (left), and immediately after initiation of a swallow (right). **(C)** VFSS Swallow rate, **(D)** VFSS Inter-swallow Interval, **(E)** VFSS Jaw Cycle:Swallow Ratio, **(F)** Jaw Excursion Rate. Each symbol indicates the average of 3–5 separate iterations for a single mouse. Mean and SD are shown. ^∗^*p* ≤ 0.05, ^∗∗^*p* ≤ 0.01, ^∗∗∗^*p* ≤ 0.001, and ^****^p ≤ 0.0001.

Analysis of total body weight revealed statistically significant differences in weight between genotype groups [*F*(3,54) = 13.76, *p* < 0.0001]. *Post hoc* tests confirmed that the Ts65Dn group weighed significantly less than both euploid control (*p* < 0.0001) and Dp(16)1Yey (*p* = 0.0006) groups (Figure 1A). There was a significant main effect of genotype on swallow rate [*F*(3,39) = 8.07], *p* = 0.0003], and *post hoc* testing indicated that the Ts65Dn group showed significantly lower swallow rates than the Dp(16)1Yey group (*p* ≤ 0.05). Inter-swallow intervals (ISI) are closely related to swallow rate, and provide a supplementary, independent measure for comparison of swallow speed differences between groups. There were corresponding significant differences between genotype groups for ISI [*F*(3,44) = 9.31, *p* < 0.0001]. *Post hoc* testing revealed that the Ts65Dn group showed significantly longer ISIs than both euploid (*p* ≤ 0.001) and Dp(16)1Yey groups (*p* ≥ 0.01). In analysis of the JSR, there were significant differences between genotypes for the number of jaw cycles per swallow [*F*(3,41) = 5.45, *p* = 0.003]. *Post hoc* testing indicated that the Ts65Dn group completed significantly more jaw cycles per swallow than both euploid (*p* ≤ 0.01) and Dp(16)1Yey groups (*p* ≥ 0.05). However, there were no significant differences between genotype groups for jaw excursion rates, [*F*(3,43) = 0.79, *p* = 0.50] (Figures 1C–F). Collectively, these analyses indicate normal jaw cycle speeds in the Ts65Dn group, but a greater number of jaw cycles preceding each swallow (JSR), associated with increases in the amount of time elapsed between swallows (ISI). That results in overall reductions in swallow rates.

#### Male and Female Ts65Dn Across an Adult Age Range

**FIGURE 2:**
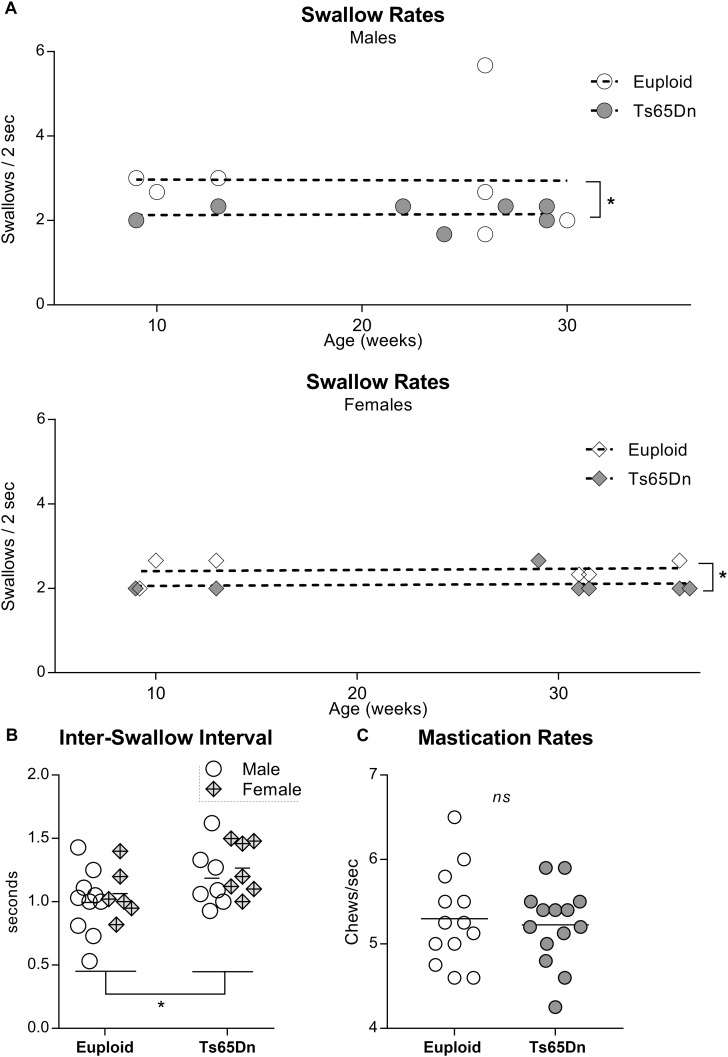
Videofluoroscopic swallow studies analysis of male and female Ts65Dn mice across an adult age range. Each symbol indicates the average of 3–5 separate iterations of the measure for a single mouse. **(A)** Swallow rates for each sex, distributed across an adult age range. **(B)** ISI measurements from mice in panel **(A)**. **(C)** Mastication rates assessed from analysis of high-speed video footage of adult male mice eating hard food pellets. ^∗^*p* ≤ 0.05.

In a separate analysis of swallow rates and ISIs across an adult age range in male and female Ts65Dn, two-way ANOVAs revealed significant main effects for genotype, but not for sex, in the absence of significant interactions between genotype and sex. Ts65Dn across an adult age range showed slower swallow rates than euploid controls [*F*(1,23) = 4.61, *p* = 0.04]. Similarly, ISIs were of significantly longer duration in the adult Ts65Dn than in the adult euploid [*F*(1,26) = 5.14, *p* = 0.03] (Figures 2A,B). Finally, video analysis of male mice eating standard hard chow pellets revealed no significant differences between Ts65Dn and euploid controls in mastication rates [*t*(25) = 0.36, *p* = 0.72] (Figure 2C).

### Tongue Muscle Analysis

#### Intrinsic Tongue Muscles in Ts65Dn and Dp(16)1Yey

**FIGURE 3:**
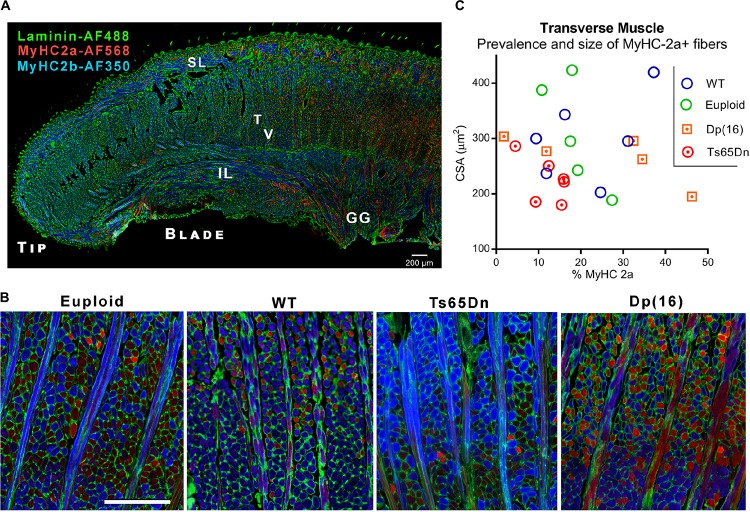
Microscopy analysis of intrinsic tongue. **(A)** Longitudinal section of the intrinsic tongue of an euploid mouse. Note the broad transition from MyHC 2b to MyHC 2a extending from the anterior tongue to the posterior tongue. SL, Superior longitudinal muscle; IL, Inferior longitudinal muscle; T, Transverse muscle; V, Verticalis muscle; GG, Genioglossus muscle. **(B)** Transverse muscle fibers (in cross section) and verticalis muscle fibers (in longitudinal section) photographed in the blade of the tongue of adult mice. Blue staining indicates myofibers positive for MyHC 2b, and red staining indicates myofibers positive for MyHC 2a. **(C)** Quantitative analysis myofiber cross-sectional area (CSA) and the percentage of fibers positive for the MyHC 2a isoform. Each symbol indicates values from one adult mouse.

Following VFSS, mice were euthanized and intrinsic tongue muscles of Ts65Dn and Dp(16) were evaluated by immunofluorescence staining and microscopy. While the majority of intrinsic tongue myofibers were positive for MyHC 2b, anterior-to-posterior anatomical distributions of MyHC isoforms were qualitatively observed in the mouse intrinsic tongue, with the greatest preponderance of MyHC 2b in the anterior tongue, and an increasing prevalence of MyHC 2a in the posterior regions of the tongue (Figure 3A). Exploratory analysis of myofiber cross-sectional area (CSA) of the transverse muscle (Figures 3B,C) revealed no significant differences between genotype groups for either CSA of MyHC 2a positive fibers [*F*(3,18) = 1.73, *p* = 0.20], nor prevalence of MyHC 2a positive fibers [*F*(3,18) = 1.44, *p* = 0.27].

#### Extrinsic Tongue MyHC Isoform Profiles Across the Adult Age Range

**FIGURE 4:**
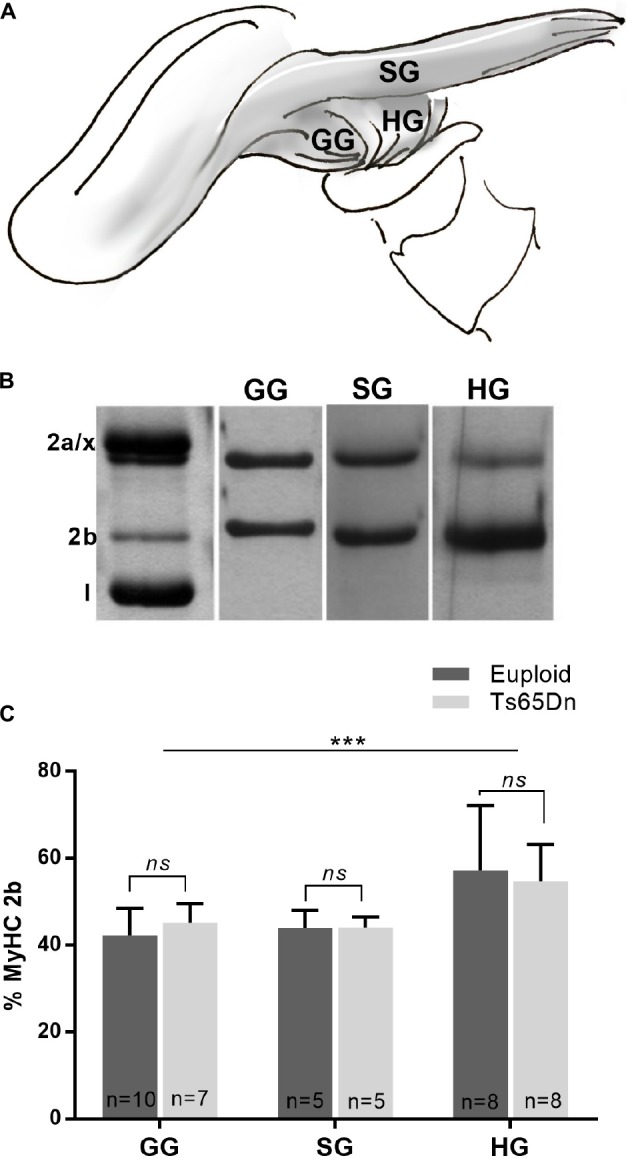
MyHC analysis of male Ts65Dn mice across an adult age range. **(A)** The styloglossus (SG), hyoglossus (HG), and genioglossus (GG) are extrinsic tongue muscles. **(B)** Extrinsic tongue muscle myosin heavy chain (MyHC) isoforms separated by SDS-PAGE and silver stained. **(C)** Relative proportion of MyHC 2b in extrinsic tongue muscle samples from mice across an adult age range. Bars indicate mean and SD. ^∗∗∗^*p* ≤ 0.001.

The genioglossus (GG) is an extrinsic tongue muscle involved in tongue protrusion, and the styloglossus (SG) and hyoglossus (HG) are involved in tongue retrusion. The GG, SG, and HG were assessed in male Ts65Dn and euploid control mice across the adult age range to assess the likelihood that genotype-specific MyHC isoform differences were present in these muscles in adult mice. No significant differences in proportion of MyHC isoforms were found between the Ts65Dn genotype and euploid controls for extrinsic tongue muscles [*F*(1,37) = 0.004, *p* = 0.95]. However, significant differences were found between extrinsic tongue muscles [*F*(2,37) = 10.35, *p* = 0.0003]. Specifically, the HG muscle had greater levels of MyHC 2b than the SG and GG muscles, across both genotypes (Figure 4).

## Discussion

A variety of feeding and swallowing challenges can occur at relatively high frequency in individuals with DS. These include reduced mastication rates (Smith et al., 2013), inefficient oral processing (Hennequin et al., 2015), and silent aspiration (aspiration of food or liquid into the trachea or lungs, in the absence of a protective cough reflex) (Jackson et al., 2016). In mice, eating involves cycles of oral processing to collect a consolidated food bolus at the region of the epiglottic vallecula, or the base of the tongue. Through food procurement and oral processing, the size of the consolidated bolus gradually increases and ultimately triggers a swallow in which the entire bolus is released from the vallecular region and proceeds through the esophagus (Figure 1B). The present study tested the hypothesis that adult mouse models of DS demonstrate abnormal feeding and swallowing performance. It was found that adult Ts65Dn swallow more slowly than Dp(16)1Yey and control mice, with longer inter-swallow interval times. Ts65Dn also showed significant increases in the JSR, which is the number of jaw cycles preceding each swallow. However, jaw excursion rates and mastication rates (measures of jaw cycle speed) were both unaffected in adult Ts65Dn. In this study, jaw excursion rates were measured by VFSS during consumption of puree, whereas mastication rates were measured by analysis of videorecordings during consumption of a hard food pellet. Therefore these were two methodologically independent measures of jaw cycle speed. The presence of typical jaw cycle speeds in Ts65Dn suggests that reduced swallow rates do not occur in the context of slower rhythmic jaw movement, but rather involve a greater number of oral movements prior to each swallow. Collectively, these differences indicate increased oral processing times prior to each swallow, and/or delayed initiation of the swallow.

The presence of increased oral processing times prior to each swallow is compatible with the possibility of inefficient oral processing, or differences in the oral movements responsible for procuring the food, forming the bolus, and conveying it to the oropharynx. If elicitation of the swallow requires a minimum bolus size and Ts65Dn mice require a greater number of oral movement cycles to achieve the requisite bolus size, delayed initiation of the swallow could result. Alternatively, a second potential cause of the difference in swallowing outcomes between genotypes could involve reduced sensitivity to the characteristics of the bolus that trigger the swallow, or increases in the threshold volume of the bolus required to trigger the swallow, as has been previously reported to occur in aging humans (Shaker et al., 1994; Miller, 2002). However, the image resolution in this VFSS study was not conducive to direct quantification of either bolus size or tongue movement. There are opportunities for future work to overcome these types of limitations through the use of VFSS systems with improved imaging capabilities, higher imaging speeds, and through supplemental experimental strategies for quantification of swallowing measures (Lever et al., 2015a; German et al., 2017).

Differences of masticatory function, and/or lingual function may influence oral processing. Prior work has identified MyHC profile differences of the digastric muscle (a muscle involved in jaw movement and chewing), in both juvenile and adult Ts65Dn (Glass and Connor, 2016). However, that work also found that juvenile Ts65Dn with atypical digastric muscle MyHC nevertheless had typical swallowing speeds as evaluated by VFSS (Glass and Connor, 2016). It is therefore possible that atypical MyHC profiles of digastric muscles in adult Ts65Dn are unlikely to be the primary cause of the significant differences in swallow rates seen in adult mice. This suggests a possibility that differences in lingual function, rather than differences in masticatory function, may be of interest as a possible factor in reduced swallowing speeds in adult Ts65Dn. Significant differences in swallow rates without the means to conclusively rule out lingual processing deficits through VFSS analysis parameters alone prompted interest in examining supplemental indicators of tongue muscle function. The extrinsic tongue muscles are responsible for tongue protrusion and retrusion. Alterations in the movements of these extrinsic tongue muscles are sometimes accompanied by alterations of MyHC isoform profiles. We evaluated the MyHC isoform profiles of these muscles to determine whether the adult Ts65Dn exhibit biochemical signatures of altered extrinsic tongue muscle function. We found that adult Ts65Dn showed no differences in MyHC isoform profiles of these muscles as compared to euploid controls. This result was similar to prior analyses of MyHC profiles of extrinsic tongue muscles in younger Ts65Dn and euploid controls at 5–6 weeks of age, which also showed no significant differences between genotypes in analysis of GG and SG MyHC (Glass and Connor, 2016). It has been previously suggested that in murine VFSS assays of drinking, jaw cycle rates are directly representative of lick rates (Lever et al., 2015b), which are accomplished through cycles of tongue protrusion (GG), and retrusion (SG and HG). Therefore, normal jaw cycle rates in Ts65Dn seen in this study, in conjunction with normal extrinsic tongue muscle MyHC profiles, provide complementary support for the conclusion that extrinsic tongue muscle function in Ts65Dn was not detectibly impaired during these VFSS assays.

The intrinsic tongue muscles (SL, IL, transverse, and verticalis) may act synergistically with the extrinsic muscles to achieve protrusion and retrusion (McClung and Goldberg, 2000), and additionally alter intrinsic tongue shape which helps to form the bolus and propel it posteriorly along the length of the tongue (Hiiemae and Palmer, 2003). However, the muscular organization of the mammalian intrinsic tongue is complex. In addition to regional MyHC profile gradients, prior studies have proposed the existence of multiple functionally distinct neuromuscular compartments spanning the length of the tongue (Mu and Sanders, 1999; Hiiemae and Palmer, 2003). While our exploratory inquiry examined the transverse muscle of the intrinsic tongue, several questions remain for future work. First, it remains to be determined whether each intrinsic tongue muscle is developmentally and anatomically typical in Ts65Dn. Secondly, incidental observation indicated a prevalence of MyHC 2b in the anterior tongue, and increasing numbers of MyHC 2a-positive myofibers in the posterior tongue (Figure 2A). To our knowledge this phenomenon has not been reported previously in mice, however, it has been reported previously in rats (Cullins and Connor, 2017). Because MyHC 2b is associated with faster contraction velocity and lower fatigue resistance as compared to MyHC 2a, (Talmadge et al., 1993; Rivero et al., 1999) it may be speculated that an anterior-to-posterior MyHC shift could have implications for tongue function. Prior studies of the intrinsic tongue in rats have found alterations in spatial gradients of MyHC profiles at older ages associated with reduced bolus speeds and reduced bolus size as determined through VFSS (Russell et al., 2013; Cullins and Connor, 2017). Because the spatial distribution of MyHC profiles in the intrinsic tongue is anticipated to be of functional significance (Stål et al., 2003), has been shown to change with aging (Cullins and Connor, 2017), and DS is associated with hallmarks of age-related functional decline (Wisniewski et al., 1978), future studies evaluating the spatiotemporal distribution of myofiber types in the intrinsic tongue in mouse models of DS may support improved understanding of functional changes in tongue movement across the lifespan in DS.

Delayed swallows in Ts65Dn are also compatible with the possibility is that Ts65Dn may experience reduced oropharyngeal sensation, which prior studies have suggested may occur in DS as evidenced by high rates of silent aspiration (Jackson et al., 2016). Impaired oropharyngeal sensation, if present, could be expected to delay swallow initiation in mice due to, among other things, reduced sensitivity to bolus size. Prior studies in mouse models of DS have reported significant reductions in innervation density of many structures (Patel et al., 2015); however, relatively little is currently known about the innervation densities of the tongue, larynx, and pharynx of Ts65Dn and Dp(16)1Yey. Studies of the peripheral innervation densities of structures involved in swallowing would conceivably be of value for efforts to identify biological mechanisms of impairment contributing to silent aspiration in DS. Similarly, it may be of interest to consider the potential role of central nervous system differences in swallowing phenotypes of Ts65Dn. For example, Ts65Dn shows a variety of GABAergic phenotypes (Deidda et al., 2015; Contestabile et al., 2017), while a recent study in typical rats has shown that GABA receptor ligands can modify murine swallowing function (Tsujimura et al., 2017). However, measures of central nervous system function were not evaluated in the present study and it is currently not known whether differences of the central nervous system impact swallowing in Ts65Dn.

While considering swallowing performance, it may be worth noting that both Ts65Dn and Dp(16)1Yey have been previously suggested to have potential utility as models of gastrointestinal abnormalities associated with DS. Ts65Dn has been suggested to have functional differences of the jejunum (Cefalu et al., 1998), and prior work in the Dp(16)1Yey model identified annular pancreas or malrotation of the intestines to be present at or below an incidence of 26% of E18.5 embryos (Li et al., 2007). It is possible, however, that pups with significant gastrointestinal abnormalities have relatively greater likelihood of perinatal or juvenile mortality. Therefore, mice with significant gastrointestinal phenotypes may or may not have been represented among the adults evaluated in the present study.

Ts65Dn and Dp(16)1Yey may in some ways offer complementary strengths and weaknesses as models of DS, however, neither model is an ideal genetic recapitulation of DS. Three different mouse chromosomes (Mmu) collectively provide regions that are syntenic to the human chromosome 21. Neither Ts65Dn nor Dp(16)1Yey comprehensively model DS through contributions from all three of these mouse chromosomes (Herault et al., 2012). Historically, Ts65Dn has been the most extensively studied mouse model of DS, and features an extra chromosome composed of a distal portion of Mmu 16, and a small centromeric portion of Mmu 17 that is not related to DS. Thus, not only does Ts65Dn incompletely model effects arising from all of the genes on Mmu 16 that are homologous to those on human chr 21, but due to contributions from unrelated genes on Mmu 17 there is an additional risk that it also may conceivably show phenotypes that are etiologically irrelevant to phenotypes of DS in humans (Gupta et al., 2016). By comparison, the Dp(16)1Yey model features a large genetic duplication that includes the full range of genes on Mmu 16 that are homologous to genes on human chr 21, thus may offer comparatively superior fidelity in modeling the gene-dosage effects in DS. However, unlike Ts65Dn, Dp(16)1Yey does not involve an extra chromosome. This bears mentioning as it has been speculated that some phenotypes of DS may be influenced by disruptions intrinsic to the presence of an additional chromosome, rather than through gene-dosage effects alone (Shapiro, 1983; Herault et al., 2012). Since Ts65Dn and Dp(16)1Yey were analyzed concurrently on the same genetic background in the present study, differences in their respective swallowing phenotypes may be attributable to functional differences in Ts65Dn caused by the contributions from Mmu 17 which are arguably not translationally relevant to DS, or may be caused by the additional partial chromosome itself in Ts65Dn, which could be of potential translational relevance to DS. Regardless, identification of swallowing phenotypes in mouse models of DS may expand opportunities for the study of dysphagia in DS to follow murine-based approaches to basic and translational research, as have been commonly used for investigations of intellectual disability in this syndrome.

Finally, it has been previously proposed that licking motor patterns in adult mice may bear some resemblance to feeding patterns in human infants (Lever et al., 2015a), in which rhythmic sucks precede each swallow (suck/swallow cycles) (Gewolb et al., 2001; Lau, 2015). The age of Ts65Dn predominately studied here (5 months) also approaches the age at which biological indicators of adult-onset neurodegeneration and age-related memory and learning impairments appear in this model (Holtzman et al., 1996; Hyde and Crnic, 2001; Hunter et al., 2003). Intriguingly, increased swallow durations characterized by increased oral processing times and delays in swallow initiation have been reported to occur in dysphagia associated with Alzheimer’s disease in the general population (Lazenby, 2008). The fact that juvenile Ts65Dn have shown normal swallow rates (Glass and Connor, 2016), while adult Ts65Dn show significantly slower swallow rates than euploid controls, suggests an age-related or maturational component to the etiology of this swallowing phenotype. Therefore, the study of feeding and swallowing in adult Ts65Dn may be anticipated to have relevance to a broad range of chronological, developmental, and biological research questions involved in feeding challenges associated with DS.

## Data Availability

The raw data supporting the conclusion of this manuscript will be made available by the authors, without undue reservation, to any qualified researcher.

## Ethics Statement

All husbandry and experimental procedures involving live mice were carried out with approval from the University of Wisconsin School of Medicine and Public Health Animal Care and Use Committee (IACUC) and were conducted in accordance with the Guide for the Care and Use of Laboratory Animals (8th ed.).

## Author Contributions

TG and NC conceived and designed the study, and contributed reagents, materials, and analysis tools. TG and LV performed the experiments and analyzed the data. TG performed the statistical analysis. All authors wrote and edited the manuscript.

## Conflict of Interest Statement

The authors declare that the research was conducted in the absence of any commercial or financial relationships that could be construed as a potential conflict of interest.
